# Impact of Deficit Irrigation on Grapevine cv. ‘Touriga Nacional’ during Three Seasons in Douro Region: An Agronomical and Metabolomics Approach

**DOI:** 10.3390/plants11060732

**Published:** 2022-03-09

**Authors:** Inês L. Cabral, António Teixeira, Arnaud Lanoue, Marianne Unlubayir, Thibaut Munsch, Joana Valente, Fernando Alves, Pedro Leal da Costa, Frank S. Rogerson, Susana M. P. Carvalho, Hernâni Gerós, Jorge Queiroz

**Affiliations:** 1 GreenUPorto—Research Centre on Sustainable Agrifood Production/Inov4Agro & DGAOT, Faculty of Sciences, Campus de Vairão, University of Porto, Rua da Agrária 747, 4485-646 Vairão, Portugal; ines.cabral@fc.up.pt (I.L.C.); susana.carvalho@fc.up.pt (S.M.P.C.); jqueiroz@fc.up.pt (J.Q.); 2 Centre of Molecular and Environmental Biology (CBMA), Department of Biology, Campus de Gualtar, University do Minho, 4710-057 Braga, Portugal; antonio.teixeira@bio.uminho.pt; 3 EA2106 Biomolécules et Biotechnologies Végétales, Université de Tours, 37200 Tours, France; arnaud.lanoue@univ-tours.fr (A.L.); marianne.unlubayir@univ-tours.fr (M.U.); thibaut.munsch@univ-tours.fr (T.M.); 4 Symington Family Estates, Vinhos SA, Travessa Barão de Forrester 86, 4431-901 Vila Nova de Gaia, Portugal; joana.valente@symington.com (J.V.); fernando.alves@symington.com (F.A.); pedro.leal.costa@symington.com (P.L.d.C.); frank.rogerson@symington.com (F.S.R.)

**Keywords:** berry composition, drought stress, grapevine yield, Mediterranean climate, metabolomics

## Abstract

The introduction of irrigation in vineyards of the Mediterranean basin is a matter of debate, in particular in those of the Douro Demarcated Region (DDR), due to the limited number of available studies. Here, we aimed to perform a robust analysis in three consecutive vintages (2018, 2019, and 2020) on the impact of deficit irrigation on the yield, berry quality traits, and metabolome of cv. ‘Touriga Nacional’. Results showed that in the peaks of extreme drought, irrigation at 30% crop evapotranspiration (ET_c_) (R30) was able to prevent a decay of up to 0.4 MPa of leaf predawn water potential (ΨPd), but irrigation at 70% ET_c_ (R70) did not translate into additional protection against drought stress. Following three seasons of irrigation, the yield was significantly improved in vines irrigated at R30, whereas irrigation at R70 positively affected the yield only in the 2020 season. Berry quality traits at harvest were not significantly changed by irrigation, except for Total Soluble Solids (TSS) in 2018. A UPLC–MS-based targeted metabolomic analysis identified eight classes of compounds, amino acids, phenolic acids, stilbenoid DP1, stilbenoid DP2, flavonols, flavan-3-ols, di-OH- and tri-OH anthocyanins, and showed that anthocyanins and phenolic acids did not change significantly with irrigation. The present study showed that deficit irrigation partially mitigated the severe summer water deficit conditions in the DDR but did not significantly change key metabolites.

## 1. Introduction

Numerous highly important wine regions in the world are located in seasonally dry areas with high evaporative demand. Climate change scenarios are predicting an increase in temperature as well as more scarce and torrential rainfall episodes. This is the case in the Mediterranean basin, including the Douro Demarcated Region (DDR). In the context in the ongoing climate change, these conditions are becoming more pronounced and may lead to negative impacts on both grapevine yield and the production of premium wines. In particular, warmer temperatures increase sugar ripeness and reduce the acidity (particularly malic acid) and flavors, resulting in unbalanced wines [1,2]. Other impacts of increasing temperatures may include the raising of potential alcohol levels [3] and a reduction in anthocyanin accumulation [4]. Phenological stages with earlier onsets of budburst, flowering, and veraison may also occur at warmer temperatures [5,6], which results in increased risks of frost damage during spring, as well as a higher incidence of grapevine-related pests and diseases [7,8,9].

Regarding drought stress, different studies have addressed the effect of water shortage on grapevine vigor, yield, and fruit quality [10,11,12,13,14,15]. It is well known that water-deficit stress can reduce the yield [16,17,18] and induce modifications of key metabolic pathways [19,20,21], shifting the abundance of transcripts and metabolites involved in phenylpropanoid, isoprenoid, carotenoid, amino acid, and fatty acid metabolism [22,23,24]. These responses, however, depend on the cultivar, crop load, vineyard age, soil type, phenological stage or canopy development [25,26,27]. In this way, irrigation may have a great influence on grapevine yield, berry quality, and wine sensory characteristics [28,29,30]. However, mild water stress can positively impact berry composition by improving sugars, flavors, and color [23,31]. In this regard, regulated deficit irrigation (RDI) has been seen as an interesting management strategy to improve productivity and berry and wine quality while saving water [32,33,34].

The vineyards of the Douro Demarcated Region (DDR), dominated by Touriga Nacional, Touriga Franca, Tinta Barroca, Tinto Cão, and Tinta Roriz (Tempranillo), are cultivated in marginal weather conditions for agricultural production [35], with soils with low water capacity holding, high evaporative demand, and low summer rainfall, making this region particularly susceptible to climate change. Touriga Nacional is considered a key Portuguese cultivar for both dry red as well as fortified wines, particularly in DDR. Although it is considered adapted to warm climate, reports of its response to water stress are debated, being classified as anisohydric [36,37] in some studies, or as isohydric in others [38]. These different responses may depend on rootstock, climate, as well as the intensity and duration of water deficits [32].

Despite being one of the most arid wine regions in the world [39], the vines of the DDR are traditionally non-irrigated. Presently, irrigation is being introduced one step at a time according to the regulations established by the Douro and Port Wines Institute, I.P (DL 7/2019), but strong scientific evidence on its benefits is still lacking. To address this issue, a robust analysis over three seasons investigated how cv. Touriga Nacional responds to specific drought conditions in the DDR, how plant response is mitigated by two different irrigation levels (R30 and R70), and how water availability affects yield, berry quality parameters, and the quantity and diversity of key primary and secondary metabolites. 

## 2. Results

### 2.1. Patterns of Water-Deficit Stress under Different Deficit Irrigation Levels

The analysis of the agrometeorological conditions in the Douro Valley wine region revealed that the 2018 season was characterized by a dry winter, followed by relatively high precipitation and low temperatures during spring and early summer (Figure S1A). High levels of precipitation were registered during March (139 mm cumulative precipitation (CP)) and June (77 mm CP), but in July and August the precipitation was scarce, with values of 3.2 and 6.2 mm, respectively. High temperatures were observed in July, August, and September with mean temperatures of 24, 27, and 24 °C, respectively. During this period, high water-deficit conditions were observed with evapotranspiration 4.0 mm above the mean. At the end of July and beginning of August one heat wave occurred with temperatures above 40 °C for six consecutive days and peaking at 44.5 °C (Figure S1A). Climatic conditions throughout 2019 season showed great variability in terms of mean temperatures. High temperatures, above 39 °C, were recorded in three days during July (11, 12, and 22 July). Of note, the hottest day of the year was recorded in July 22 (41.6 °C).

In general, precipitation was lower in 2019 than in 2018 (Figures S1 and S2), but in July and August of 2019 precipitation was higher and temperatures were lower, so vines suffered from less drought stress during summer (see below). Throughout 2020, climatic conditions revealed some fluctuations in temperature and precipitation values (Figure S3). In June an abrupt rise in temperatures was registered, particularly between the 22nd and 23rd, with temperatures of 38.5 and 40.4 °C, respectively. As in the two previous years, by the time of harvest (September) the precipitation was relatively low. In general, the year of 2020 was the least rainy of the three, with a total rainfall of 33.9 mm CP, and the hottest, with an average temperature of 16.7 °C, followed by the year 2019 (15.9 °C) and the 2018 vintage (15.7 °C).

**Figure 1:**
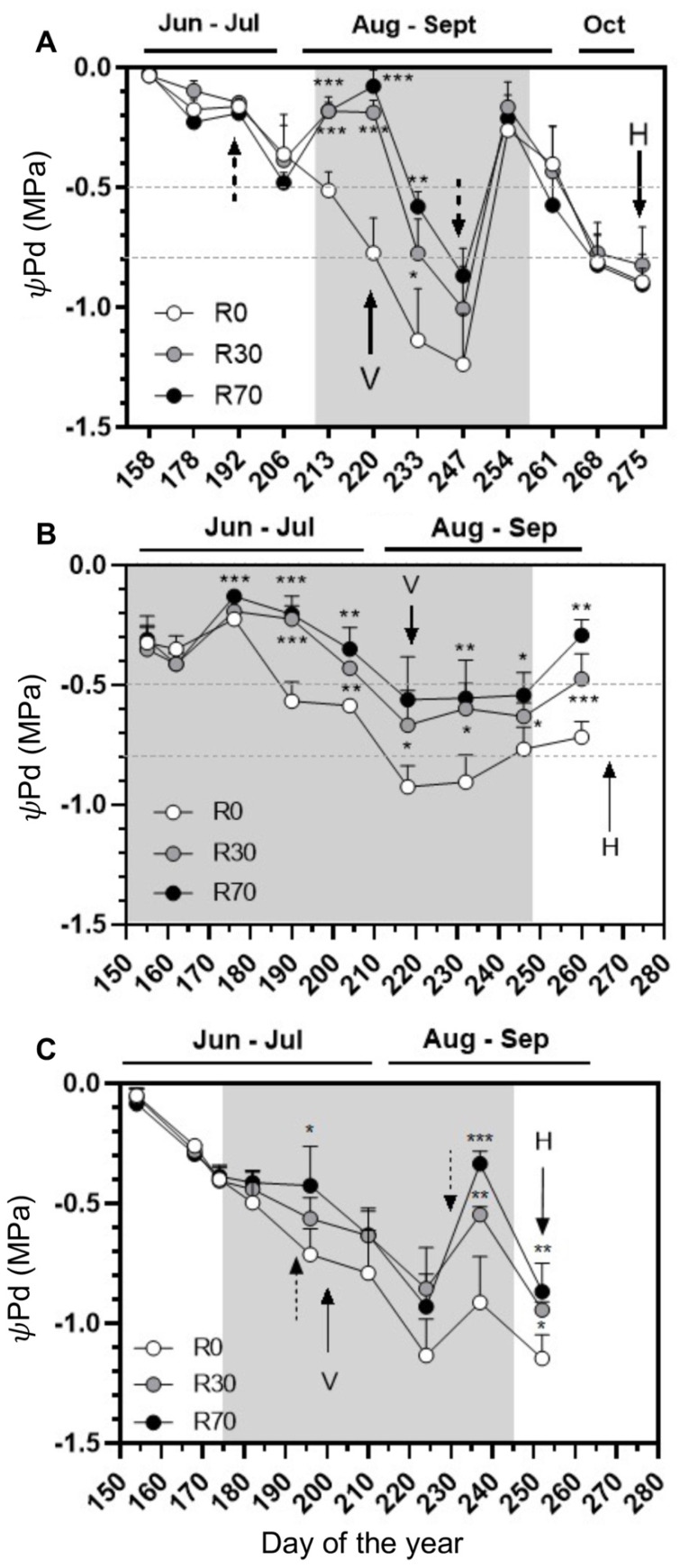
Predawn leaf water potential (ΨPd) of grape cv. ‘Touriga Nacional’ in different irrigation conditions, during (**A**) 2018, (**B**) 2019, and (**C**) 2020 growing seasons. Irrigation conditions: R0 = non-irrigated plants; R30 = deficit irrigation corresponding to 30% of evapotranspiration; R70 = deficit irrigation corresponding to 70% of evapotranspiration. V indicates veraison and H harvest. Dotted arrows indicate dates of precipitation (see supplementary data), shadowed vertical band corresponds to the irrigation interval. The dotted horizontal band represents moderate to severe (−0.5 to −0.8 MPa) water deficit. Results represent mean ± SD of four replicates and the values are marked with asterisks to denote the significance level as compared to the control: * *p* ≤ 0.05; ** *p* ≤ 0.01; *** *p* ≤ 0.001.

In the 2018 season, predawn leaf water potential (ΨPd), monitored from fruit set (DOY 155) until harvest (DOY 279), every 5–15 days, showed severe water-deficit stress episodes at the end of August in all treatments, i.e., values below −0.8 MPa according the scale [25]. After the onset of irrigation (DOY 206), the ΨPd steadily increased from −0.5 to ca. −0.2 MPa followed by a decrease from pre- to post-veraison until −0.9 Mpa at 30% ET_c_ (R30) (Figure 1A). In non-irrigated vines, ΨPd gradually decreased from moderate (−0.5 Mpa) to severe (−1.2 Mpa) until the beginning of September (DOY 247). Under these extreme drought conditions, irrigation at R30 and R70 (70% ET_c_) clearly mitigated the impact of water deprivation, although no significant differences were observed in this parameter when comparing these two irrigation levels (Figure 1A). Still in 2018, a severe precipitation event occurred at DOY 248 (26 mm, September 5), promoting a steep increase in the ΨPd up to ca. −0.2 Mpa both in control and irrigated vines, followed by a decrease until harvest (DOY 275), when the ΨPd of non-irrigated and irrigated vines (irrigation stopped at DOY 254) reached −0.8 Mpa, indicating severe drought stress. From this point until harvest, no significant differences were found between R0, R30, and R70 (Figure 1A).

In 2019, ΨPd was monitored from DOY 155 (13 days after fruitset) until DOY 260 (8 days before harvest). After the onset of irrigation, ΨPd values increased until DOY 176 (at both R30 and R70) and then steadily decreased until veraison, when non-irrigated vines suffered from severe drought stress (Figure 1B). A steady decrease in drought stress of both irrigated and non-irrigated vines coincided with the occurrence of precipitation after the DOY 218 until harvest (Figure 1B). However, at harvest, non-irrigated vines suffered from moderate water stress (ΨPd below −0.5 Mpa).

In 2020, ΨPd was monitored from 2 June (DOY 154), two weeks previous to veraison, until 8 September (DOY 252), the harvest date. After the onset of irrigation (DOY 174), there was a decrease in the values of ΨPd, more evident in non-irrigated vines. By mid-August, extreme drought conditions were registered and irrigation at R30 and R70 clearly mitigated the impact of water deprivation (Figure 1C). At the time of the harvest, vines were under drought stress, as in the 2018 vintage, and R30 and R70 partially mitigated water deprivation.

### 2.2. Effect of Deficit Irrigation on Grapevine Yield, Vigor, and Berry Quality 

**Figure 2:**
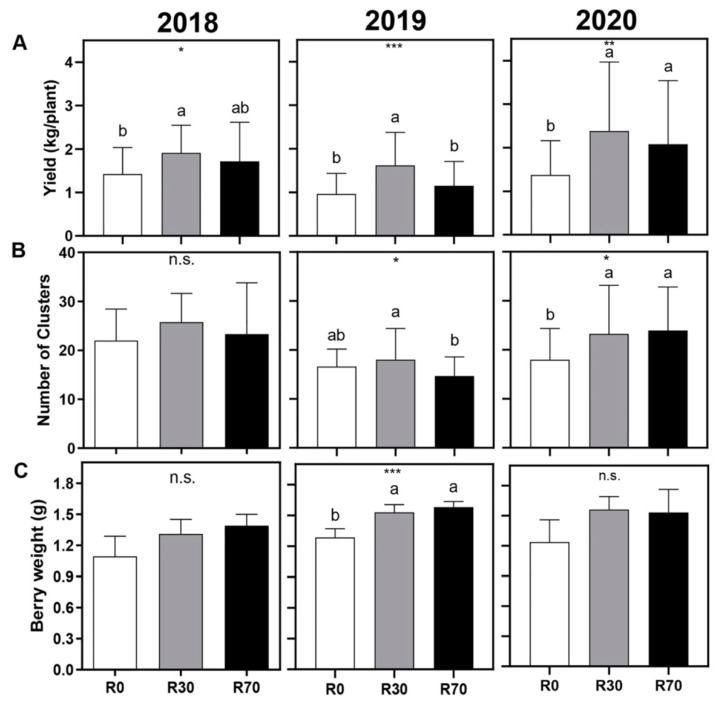
Effect of different irrigation conditions on (**A**) yield (kg/plant), (**B**) number of clusters per plant, and (**C**) berry weight, in 2018, 2019, and 2020 vintages of grapevine cv. ‘Touriga Nacional’. Irrigation conditions: R0 = non-irrigated plants; R30 = deficit irrigation corresponding to 30% of evapotranspiration; R70 = deficit irrigation corresponding to 70% of evapotranspiration. Results represent mean ± SD of four replicates. Asterisks indicate ANOVA statistical differences: * *p* ≤ 0.05; ** *p* ≤ 0.01; *** *p* ≤ 0.001; n.s. = non-significant. Lowercase letters indicate differences between treatments.

In the three vintages (2018, 2019, and 2020), R30 promoted a significant (*p* values = 0.023, 0.001, and 0.009, respectively) and consistent increase in yield (kg/plant) by 34, 69, and 73%, respectively, while R70 only increased the yield in 2020 (by 51%) as compared to non-irrigated plants (Figure 2A). In 2019 and 2020, but not in 2018, higher number of clusters were produced by vines irrigated at R30 (16 and 29%, respectively). R70 promoted a higher number of clusters only in the 2020 season (Figure 2B). Regarding the mean berry weight at harvest, differences were not statistically significant between treatments in 2018 and 2020, while in 2019 the berry weight was 16 and 19% higher at R30 and R70, respectively, compared with control vines (Figure 2C).

**Figure 3:**
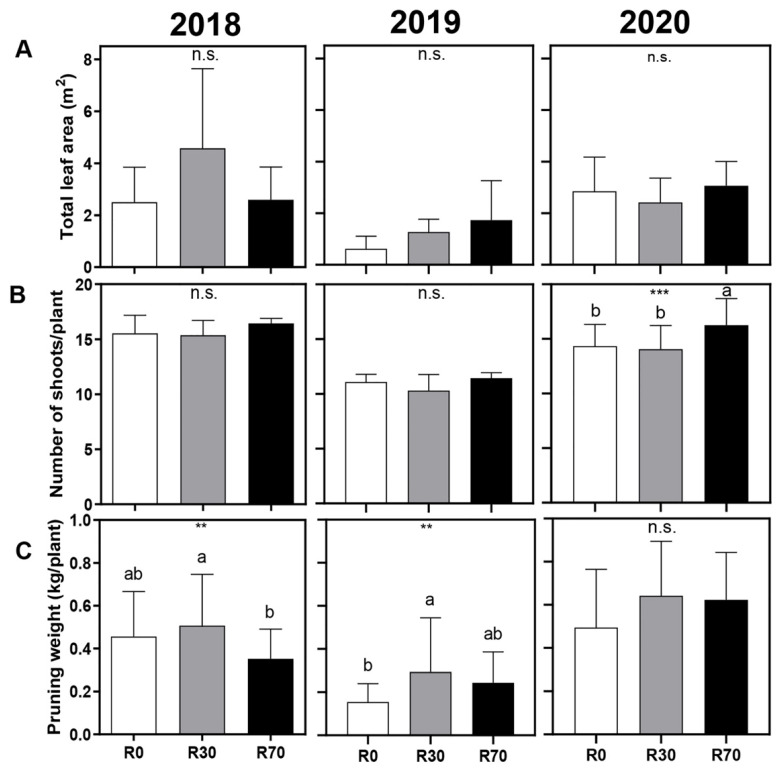
Effect of different irrigation conditions on (**A**) total leaf area at harvest, (**B**) number of shoots per plant, and (**C**) pruning weight at dormancy stage, in 2018, 2019, and 2020 vintages of grape cv. ‘Touriga Nacional’. Irrigation conditions: R0 = non-irrigated plants; R30 = deficit irrigation corresponding to 30% of evapotranspiration; R70 = deficit irrigation corresponding to 70% of evapotranspiration. Results represent mean ± SD of four replicates. Asterisks indicate ANOVA statistical differences: ** *p* ≤ 0.01; *** *p* ≤ 0.001; n.s. = non–significant. Lowercase letters indicate differences between treatments.

Concerning the effect of irrigation on plant vigor parameters, no significant differences were found on total leaf area at harvest in the three seasons (Figure 3A). A significant increase was observed in shoots per plant only at R70, in the 2020 season (Figure 3B, Tables S5 and S6). Pruning weight, registered during winter pruning, showed an increase in plants irrigated at R30, but this effect was only significant in the 2019 vintages (Figure 3C, Tables S3 and S4). In general, an additional level of irrigation (R70) did not significantly impact plant vigor.

**Figure 4:**
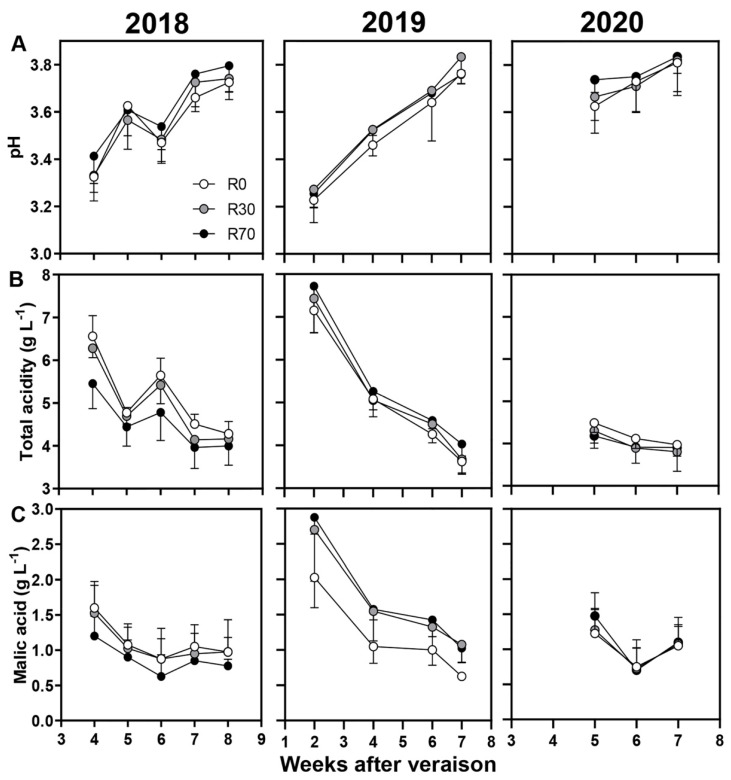
Berry quality attributes during maturation until harvest: (**A**) pH, (**B**) total acidity, and (**C**) malic acid in 2018, 2019, and 2020 vintages of grape cv. ‘Touriga Nacional’ in different irrigation conditions. Irrigation conditions: R0 = non—irrigated plants; R30 = deficit irrigation corresponding to 30% of evapotranspiration; R70 = deficit irrigation corresponding to 70% of evapotranspiration. Results represent mean ± SD of four replicates.

**Figure 5:**
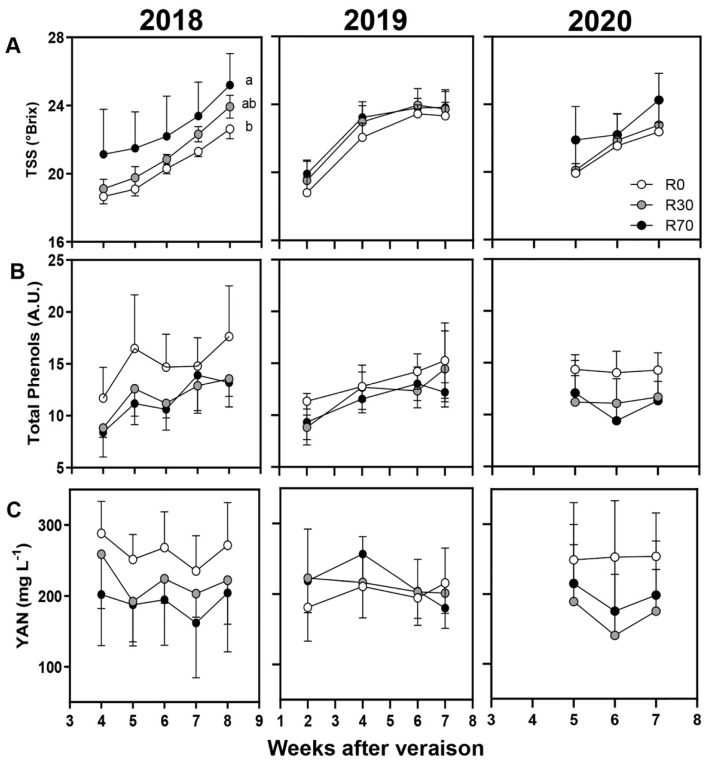
Berry quality attributes during maturation until harvest: (**A**) Total Soluble Solids (TSS), (**B**) total phenols (A.U.—arbitrary units), and (**C**) yeast assimilable nitrogen (YAN) in 2018, 2019, and 2020 vintages of grape cv. ‘Touriga Nacional’ in different irrigation conditions. Irrigation conditions: R0 = non—irrigated plants; R30 = deficit irrigation corresponding to 30% of evapotranspiration; R70 = deficit irrigation corresponding to 70% of evapotranspiration. Results represent mean ± SD of four replicates. Lowercase letters indicate differences between treatments.

Berry quality attributes, including pH, TSS (°Brix), total acidity, malic acid, total phenols, and YAN were also analyzed in non-irrigated and irrigated conditions in 2018, 2019, and 2020 (Figure 4 and Figure 5). For all attributes tested, no significant differences at harvest, except for TSS in 2018 (Tables S1 and S2) and malic acid in 2019, were observed. Both TSS and malic acid values were consistently higher at R70 compared to R0 and R30 during maturation, but significant differences were observed only at harvest between R70 and R0 (25 and 22 Brix; 0.63 and 1.03 g L^−1^, respectively) (Figure 4A,C, Table S1) possibly due to two important precipitation events during this phase.

### 2.3. Effect of Deficit Irrigation on Berry Metabolome

For a deeper investigation on the impact of the two irrigation levels on berry metabolism, UPLC–MS-based targeted metabolomic analysis was performed, as previously described [40,41] on grape berry extracts from 2018, 2019, and 2020 seasons. Forty-four compounds were identified, including: 6 amino acids, 5 phenolic acids, 2 stilbenoids DP1, 2 stilbenoids DP2, 7 flavonols, 11 flavan-3-ols, 3 di-OH anthocyanins, and 8 tri-OH anthocyanins. Raw data are presented in Table S7.

**Figure 6:**
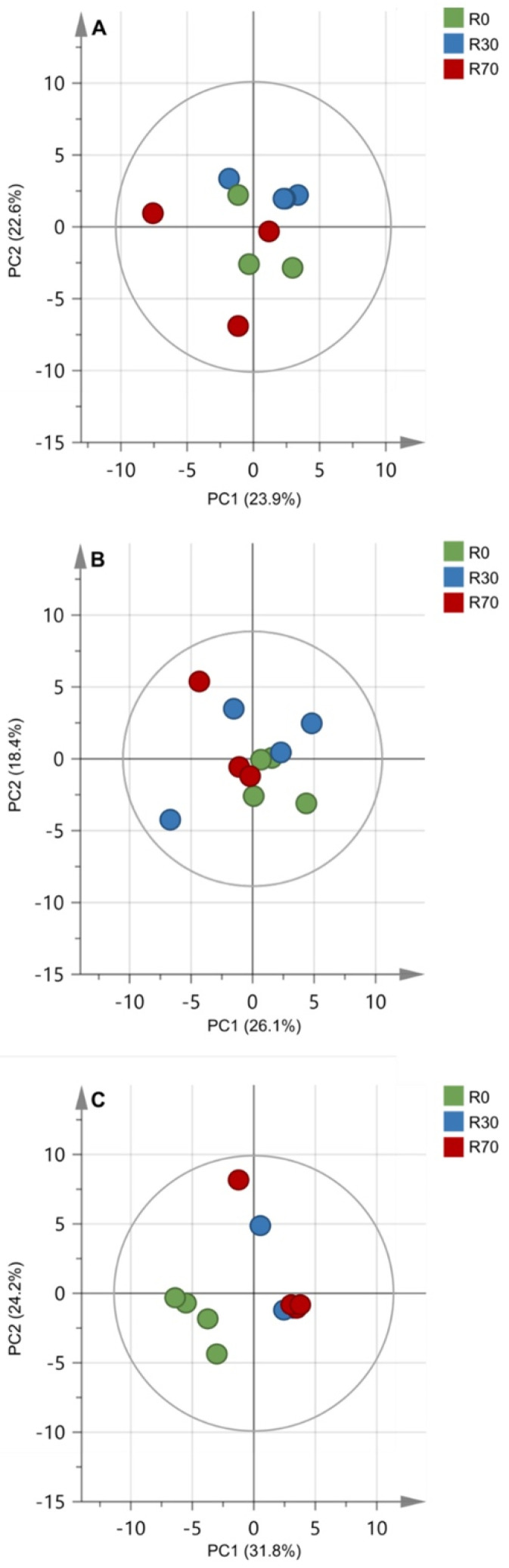
Principal component analysis of mature grape berry metabolic compounds in three vintages, 2018 (**A**), 2019 (**B**), and 2020 (**C**). Score plots of UPLC–MS-based metabolomic data from ethanolic extracts of berries under different irrigation treatments: R0 = non—irrigated plants; R30 = deficit irrigation corresponding to 30% of evapotranspiration; R70 = deficit irrigation corresponding to 70% of evapotranspiration.

The PCA score plots of the two first components explained 56% of variation found in the 2020 season concerning the metabolic compounds of the mature grape berry, whereas in the other seasons the percentage of variance explained was lower (46.5% in 2018 and 44.5% in 2019) (Figure 6). Interestingly, in 2020 it was possible to discriminate control plants with no irrigation from irrigated plants (Figure 6C), whereas no clear discrimination was observed on 2018 and 2019 seasons (Figure 6A,B). Nonetheless, even in 2020 the two groups of irrigated plants, R30 and R70, could not be discriminated.

**Figure 7:**
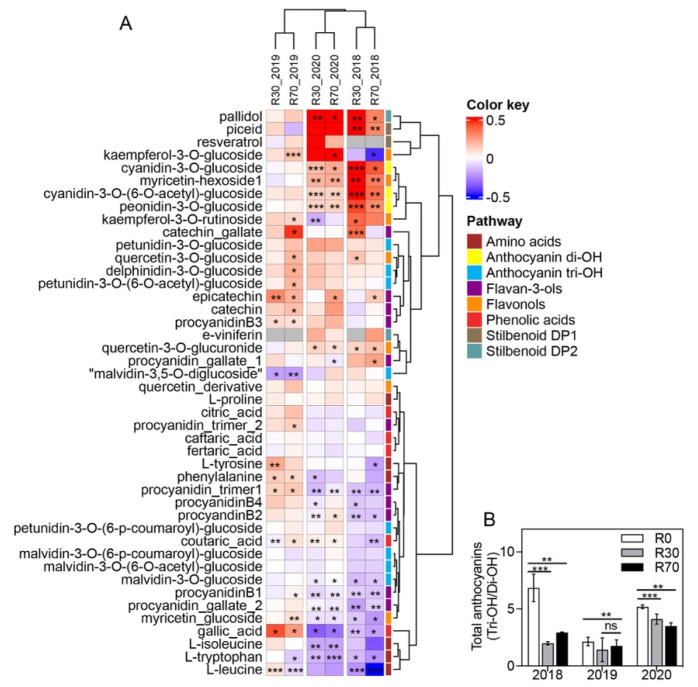
Metabolite changes in mature grape berries from Douro region subjected to different irrigation conditions in 2018, 2019, and 2020 vintages. (**A**) Heatmap shows the levels of individual amino acids, phenolic acids, stilbenoids, flavonols, flavan–3–ols, anthocyanins Di–OH, and anthocyanins Tri–OH. Values are the logarithmic transformed fold change (R30/R0 and R70/R0) of berry compounds from Touriga Nacional. Columns represent the means ± SD (*n* = 4). (**B**) Ratio of total Tri–OH/Di–OH anthocyanins for the three vintages. Asterisks denote the significance levels as comparing R30 to R0 and R70 to R0) of berry compounds and anthocyanins ratio: * *p* ≤ 0.05; ** *p* ≤ 0.01; *** *p* ≤ 0.001; ns = non–significant.

A heatmap representation of each metabolite change in R30 and R70 compared to R0 is shown in Figure 7. R30 irrigation significantly increased only three amino acids (L-leucine, L-tyrosine, and L-phenylalanine) in the 2019 vintage, while a decrease was observed in most of the detected amino acids in 2018 and 2020 vintages (Figure 7A).

Regarding phenolic acids, R30 and R70 promoted a significant increase in gallic acid in the 2019 vintage (62 and 82%, respectively) and a decrease in the other two vintages (up to 54% from control). Coutaric acid increased in the 2020 vintage for both R30 and R70 (97 and 91%, respectively) but decreased in both irrigation treatments of 2018. 

Five flavonols (kaempferol-3-*O*-glucoside, myricetin-hexoside1, quercetin-3-*O*-glucoside, kaempferol-3-*O*-rutinoside, and quercetin-3-*O*-glucuronide) increased in two of the three vintages, while myricetin glucoside decreased (up to 49%) in both 2018 and 2020 vintages for both irrigation treatments (Figure 7A). Likewise, from the 11 different flavan-3-ols identified, up to six significantly decreased in grapes subjected to R30 or R70 in the 2018 and 2020 vintages, while in general flavan-3-ols increased in the 2019 vintage (Figure 7A). Regarding anthocyanins, irrigation significantly increased some Tri-OH: delphinidin-3-*O*-glucoside, petunidin-3-*O*-(6-*O*-acetyl)-glucoside at R70 in the 2019 vintage. Similarly, Di-OH anthocyanins’ significant increase was observed on both R30 and R70 irrigations in the 2018 and 2020 vintages: cyanidin-3-*O*-glucoside, cyanidin-3-*O*-(6-*O*-acetyl)-glucoside, peonidin-3-*O*-glucoside, while most of the Tri-OH decreased in response to both R30 and R70 in these vintages: petunidin-3-*O*-(6-p-coumaroyl)-glucoside, malvidin-3-*O*-(6-p-coumaroyl)-glucoside, malvidin-3-*O*-(6-*O*-acetyl)-glucoside, malvidin-3-*O*-glucoside (Figure 7A). The ratio between Tri-OH and Di-OH anthocyanins indicated a significant decrease in the former in both irrigation treatments (Figure 7B).

Contrary to phenolics, the stilbenoids DP1 E-piceatannol and E-piceid increased at R30, while at R70 only E-piceid increased. Among stilbenoids DP2, only pallidol was significantly increased at both R30 and R70 treatments in the 2018 and 2020 vintages (Figure 7A).

## 3. Discussion

### 3.1. Irrigation at R30 and R70 Did Not Substantially Change Berry Quality Traits

The damaging effects of heat and water deficit stress and the potential benefits of deficit irrigation have been widely reported in grapevines (for a review see [15,42,43,44]). The meteorological conditions of 2018 and 2020 seasons in Douro region were similar, but fewer extreme events were reported in the latter. Remarkably, September 2018 was the warmest month of the last 40 years [45]. The 2019 season was characterized by mild temperatures throughout the year. It is well known that heat combined with a severe water-deficit stress may compromise photosynthesis [32,46,47,48,49,50,51,52,53]. Other consequences of supra-optimal temperatures under conditions of water scarcity may include source–sink imbalance and incomplete berry maturation [19,32,54,55], thus, we expected significant changes in the berry composition in irrigated Touriga Nacional vines. However, both levels of irrigation did not translate into significant changes in berry quality traits including Brix, pH, total phenolics, total anthocyanins, and phenolic acids (Tables S1, S3 and S5, Figure 7A). This behavior and other responses observed in the present study may correlate with the plant capacity to adjust stomatal conductance, which controls water loss and surface temperature under water-deficit stress. In this regard, new studies in grape varieties with different tolerance to drought stress may allow clarifying the results of the present paper.

From the end of July until the end of August of 2018 and 2020, under extreme drought conditions, both R30 and R70 prevented a decay of up to 0.37 units of leaf ΨPd when ΨPd of non-irrigated plants reached values up to –1.2 MPa, at which they were far below the hydric comfort (Figure 1). The observed positive effects of irrigation on yield at harvest are in line with previous reports [20,33,42,44,55,56], but more water (irrigation at R70) did not translate into additional gains in plant growth and productivity (Figure 2 and Figure 3), possibly due to a low soil holding water capacity. Accordingly, plant water status under R30 and R70 was equivalent in each season during the irrigation periods.

In the three vintages of 2018, 2019, and 2020, the Brix at harvest did not change when irrigation was applied either at R30 or R70 (Figure 5). The effect of water deficit on grape berry sugar content is dependent on the genotype and on the developmental stage [23,28,57,58]. Again, both irrigation levels R30 and R70 did not significantly affect the acidity parameters of the berries at harvest (Figure 4), although it has been reported that the titratable acidity in grapes from vines under deficit irrigation is reduced [53,58,59,60] and that the malate/tartrate ratio is in general lower in vines with low water status due to malate breakdown [10,32].

### 3.2. A Targeted Metabolomic Analysis Showed Modifications in the Relative Abundance of Primary and Secondary Metabolites in Response to R30 and R70

It has been reported that the water status modulates secondary metabolism (reviewed by [14]). These prompted us to perform a targeted metabolomics analysis to evaluate in more detail changes in specific primary and secondary metabolites in response to irrigation. In a previous study, high temperature at mid-ripening coupled with moderate deficit irrigation (25% of ET_c_) reduced total anthocyanin content, possibly by degrading these compounds or/and inhibiting their biosynthesis [44,61]. Although in the three consecutive seasons of 2018, 2019, and 2020 irrigation did not substantially affect the majority of berry quality attributes, the metabolomics analysis showed that irrigation at R30 and R70 differentially affected the composition of some key metabolites, including amino acids, phenolic acids, stilbenoids, flavonols, flavan-3-ols, and anthocyanins of Touriga Nacional grapes harvested in 2018 and 2020 (Figure 7). The majority of the amino acids analyzed decreased under irrigation (except phenylalanine in 2019), together with phenolic acids. Proline acts as energy source, an antioxidant, an osmoprotectant, and contributes to the sweet taste to the berries [62,63]. We observed that this amino acid, which normally increases in water-stressed plants, did not suffer alterations in berries at harvest subjected to R30 and R70 in all three vintages. 

Conflicting results have been reported in the literature regarding the effect of water deficit on stilbenoids synthesis (reviewed by Teixeira et al., 2013 [14]). Thus, while a short effect of drought was observed on resveratrol concentration in grape berry skin in Barbera cultivar [64], a substantial increase in mRNA abundance of steroid sulfatase (STS) was observed in Cabernet Sauvignon cultivar [65]. On the contrary, in the present study both R30 and R70 stimulated stilbenoids synthesis in Touriga Nacional in 2018 and 2020 vintages with more severe temperature and drought conditions. In addition, resveratrol synthesis was not stimulated during the trial.

Although total anthocyanins did not change with irrigation, an increase in the di-OH anthocyanins in response to R30 and R70 was observed in 2018 and 2020, while both irrigation protocols reduced only one tri-OH anthocyanin in those vintages. It has been shown that drought conditions stimulate anthocyanin hydroxylation and methoxylation of the flavonoid B-ring [62]. In contrast, high temperature reduced anthocyanin hydroxylation in grape berries [44,66]. Trihydroxylated anthocyanins (delphinidin, petunidin, and malvidin-3-glucosides) are more stable in wines than dihydroxylated ones (cyanidin and peonidin-3-glucosides) [67]. Color of anthocyanins changes progressively from red to blue as tri-OH/di-OH anthocyanin ratio increases during ripening [62]. The present study indicated that irrigation has a positive effect on di-OH anthocyanins (cyanidin-3-*O*-glucosid; peonidin-3-*O*-glucosid) and stilbenoids (pallidol; piceid) and a negative effect on amino acids (L-isoleucine; L-tryptophan) and flavan-3-ols (procyanidins). These significant effects were observed in the vintages with more severe climate conditions, although PCA analysis of berry metabolites revealed that only in 2020 irrigation was able to explain the variation between the variables non-irrigated and irrigated, but not between R30 and R70 (Figure 6).

## 4. Materials and Methods

### 4.1. Field Conditions and Experimental Design

The experimental trial was conducted during 2018, 2019, and 2020 in a commercial vineyard with a sandy loam soil located in Douro Superior sub-region, Portugal (41°14′36″ N, 7°06′55″ W), at an altitude of about 140 m. Touriga Nacional cultivar (*Vitis vinifera* L.) used in this study was planted in 2014, being grafted in 196–17 Cl rootstock. The rows of vines were oriented in west southwest to east/northeast, spaced at 2.2 m between rows, 1.0 m along the row, and trained on a vertical shoot position trellis system, uniformly pruned on a unilateral Royat cordon, ca. 10 buds per vine. The cordon was 0.5 m above soil. During growing season, three irrigation treatments were imposed: non-irrigated plants, corresponding to the control (CTRL; R0), irrigated plants corresponding to 30% of crop evapotranspiration (ET_c_; R30), and irrigated plants corresponding to 70% ET_c_ (R70). The experimental set-up was a complete randomized block design, containing four blocks, with five border plants between each block to avoid watering interference. Each block was composed by two rows of plants comprising eight vines as treatments. A total of 96 plants were used (4 blocks × 3 block combination × 8 experimental units). This experimental set-up is part of the demo site related to the VISCA Project (VISCA—Vineyards’ Integrated Smart Climate Application H2020/Research and Innovation action; Grant Agreement no. 730253).

### 4.2. Irrigation

Irrigation was performed using the drip-irrigation method, composed of pipelines installed along the plant rows and 0.5 m above the soil with drippers spaced at 0.5 m (2 per vine). The irrigation flow rate supplied was 3.6 L h^−1^ in R30 and 7.2 L h^−1^ in R70. The reference evapotranspiration (ET0) per week was calculated applying the Penman–Monteith equation [68]. The ET0 was used, along with a constant crop coefficient (Kc = 0.7) to calculate the amount of water required by plants (ET_c_), using the equation ET_c_ = Kc × ET0. Precipitation was subtracted from ET_c_ each week, and the calculated amount of water required was applied the following week. The constant Kc was chosen from previous studies, considering the months where irrigation occurred, the vineyard characteristics, and the values described in the literature [69]. Irrigation started when values of pre-dawn leaf water potential (Ψpd) reached ca. −0.4 MPa, which indicated a weak to moderate water deficit [65]. Water was supplied every week, starting on 25 July (DOY 206) and ending three weeks before harvest (DOY 275) in 2018. In 2019, irrigation started on 28th May (DOY 148) and ended at DOY 248 (5th September), two weeks before harvest. In 2020 irrigation started on 23 July (DOY 175) and ended at DOY 245 (1 September), three weeks before harvest, due to precipitation in the week before harvest.

### 4.3. Meteorological Data

Meteorological data were obtained from an automatic weather station (ADCON, Kempten, Germany) located in the experimental site. Data on the precipitation (P), maximum temperature (Tmax), minimum temperature (Tmin), average temperature (Tavg), radiation (Insol), and evapotranspiration (Etp) were computed. Day of the year (DOY) was also calculated. 

### 4.4. Grapevine Water Status Determination

Pre-dawn leaf water potential (Ψpd) was measured using a Schölander pressure chamber (PMS Instruments Co., Model 600, Corvallis, OR, USA) [70], throughout the growing season, from fruit set until harvest and performed 2 h before sunrise. The measurements were carried out in eight plants per treatment (using one well exposed and fully expanded leaf per plant), every 7–15 days according to climatic conditions and the phenological stage of the plant. Irrigation was carried out one day after this measurement, during the night.

### 4.5. Phenological Stages and Vegetative Growth

Phenological stages (including budbreak, fruit set, and veraison) were recorded when 50% of the plants within each experimental unit reached that stage. At harvest, total leaf area was registered using the method developed by Lopes and Pinto (2005) [71]. At dormancy stage, the number of shoots was recorded, vines were pruned, and pruning mass (kg/vine) was individually determined for each vine. The number of shoots per plant and pruning weight were recorded at the end of each season, after leaf fall.

### 4.6. Yield Parameters and Berry Composition during Development

To evaluate the harvest parameters, yield per plant (g) and the number of bunches were measured. For berry composition analysis during the growing cycle, a sample of 50 berries per replication (i.e., per block, resulting in 200 berries per treatment) was collected weekly starting on the fourth week after veraison (WAV) until harvest (8 WAV) in 2018. In 2019 vintage, berries were collected from the second week after veraison until harvest (2 WAV). In 2020 season, berries were collected from 5 WAV, until 7 WAV, harvest day. Berry samples were obtained from ± 6 bunches of each plant and selected from different bunch positions. The berries were crushed, and several biochemical quality parameters were determined: pH, total soluble solids (TSS; Brix), total acidity (g L^−1^), malic acid (g L^−1^), total phenolics (absorption wave length of 280 nm; absorption unit—A.U.) and yeast assimilable nitrogen (YAN; mg L^−1^) using OenoFossTM (FOSS Analytical, Hilleroed, Denmark) according to the manufacturer’s protocol equipment and by official methods of the Organisation Internationale de la Vigne et du Vin (OIV, https://www.oiv.int/, 2014).

### 4.7. Biochemical Analysis of Mature Berries

At harvest, 12 berries per replicate were collected totalizing 48 berries in each treatment (3 berries × 4 plants × 4 replicates). Samples were immediately frozen and stored at −80 °C for further analysis. Berries from each block were pooled and grounded with liquid nitrogen to a fine powder and freeze dried in Christ Alpha 2–4 LD Plus lyophilizer (Sigma –Aldrich^®^, Darmstadt, Germany) to be used in several biochemical quantification assays.

### 4.8. UPLC–MS-Based Metabolic Profiling

Methods for metabolic profiling of grape berries were adapted from previous studies [40,41]. An extract using 50 mg of berry dry weight (D.W.) and 1 mL of 80% (*v*/*v*) methanol was prepared in closed Eppendorf tubes. After 30 min of sonication, samples were macerated overnight at 4 °C in the dark and centrifuged at 18,000× *g* for 10 min. The supernatant was diluted 5-fold in 80% (*v*/*v*) methanol and stored at −20 °C prior to further analyses. UPLC–MS was performed using an ACQUITY™ Ultra Performance Liquid Chromatography system coupled to a photo diode array detector (PDA) and a Xevo TQD mass spectrometer (Waters, Milford, MA, USA) equipped with an electrospray ionization (ESI) source controlled by Masslynx 4.1 software (Waters, Milford, MA, USA). Analyte separation was achieved by using a Waters Acquity HSS T3 C18 column (150 × 2.1 mm, 1.8 μm) with a flow rate of 0.4 mL min^−1^ at 55 °C. The injection volume was 5 μL. The mobile phase consisted of solvent A (0.1% formic acid in water) and solvent B (0.1% formic acid in acetonitrile). Chromatographic separation was achieved using an 18 min linear gradient from 5 to 50% solvent B. MS detection was performed in both positive and negative modes. The capillary voltage was 3000 V and sample cone voltages were 30 and 50 V. The cone and desolvation gas flow rates were 60 and 800 Lh^−1^. Identification of analytes was based on retention times, *m*/*z* values, and UV spectra and by comparison with commercial standards, own purified compounds, or data from literature when no authentic standards were available. The complete description of analyte identification can be seen in [41] and the present ID numbers are L-proline (m0), L-leucine (m1), L-isoleucine (m2), phenylalanine (m3), L-tyrosine (m4), L-tryptophan (m5), cyanidin-3-*O*-glucoside (m6), peonidin-3-*O*-glucoside (m7), cyanidin-3-*O*-(6-*O*-acetyl)-glucoside (m8), delphinidin-3-*O*-glucoside (m9), petunidin-3-*O*-glucoside (m10), malvidin-3-*O*-glucoside (m11), petunidin-3-*O*-(6-*O*-acetyl)-glucoside (m12), malvidin-3-*O*-(6-*O*-acetyl)-glucoside (m13), petunidin-3-*O*-(6-p-coumaroyl)-glucoside (m14), malvidin-3-*O*-(6-p-coumaroyl)-glucoside (m15), malvidin-3,5-*O*-diglucoside (m16), catechin (m17), epicatechin (m18), catechin gallate (m19), procyanidinB1 (m20), procyandinB2 (m21), procyanidinB3 (m22), procyanidinB4 (m23), procyanidin gallate 1 (m24), procyanidin trimer 2 (m25), procyanidin gallate 2 (m26), procyanidin trimer1 (m27), kaempferol-3-*O*-glucoside (m28), quercetin-3-*O*-glucoside (m29), quercetin-3-*O*-glucuronide (m30), myricetin-hexoside1 (m31), myricetin glucoside (m32), quercetin derivative (m33), kaempferol-3-*O*-rutinoside (m34), gallic acid (m35), citric acid (m36), coutaric acid (m37), caftaric acid (m38), fertaric acid (m39), resveratrol (m40), piceid (m41), pallidol (m42), e-viniferin (m43). Extraction and UPLC–MS analyses were performed in quadruplicates.

### 4.9. Data Mining

UPLC–MS analyses were achieved using selected ion monitoring (SIM) mode and resulting SIM chromatograms were integrated using the subroutine QuanLynx 4.1 for data mining. A pool of all samples was prepared to obtain a quality control sample (QC) and the samples were randomly injected independently from treatment conditions. Three QC samples were injected at the beginning of the sample set and one QC sample was injected every eight samples to check for potential analytical drifts. QC samples were analyzed by Principal Component Analysis to evaluate the reproducibility of the UPLC–MS method [72].

### 4.10. Statistical Analysis

All data are presented as mean values ± standard deviation (SD) of four replicates per block in each assay. Each treatment included 4 blocks of 8 biological replicates. For the berry composition and metabolomic analysis, berries from the eight vines per block were pulled, according to the description above. Results were compared with one-way ANOVA using Prism 7.0 (GraphPad Software, Inc., La Jolla, CA, USA). The Principal Component Analyses were undertaken using SIMCA P + version 12.0 (Umetrics AB, Umeå, Sweden) and heatmap metabolomics was performed with the ComplexHeatmap package (v1.18.1) on Bioconductor v3.9 after value normalization by using the R center and scale functions.

## 5. Conclusions

To the best of our knowledge, this is the first robust study (performed in three consecutive seasons) integrating meteorological conditions with agronomical, analytical, and metabolomic data in vines of Douro Region under different irrigation levels (R30 and R70). In mid-summer, R30 was able to prevent a decay of up to 0.4 MPa of leaf predawn water potential and improved plant productivity, while R70 did not translate in additional protection against drought stress, possibly due to a low water capacity holding of the soil. Moreover, both irrigation levels did not significantly change important berry quality traits including Brix, pH, total phenolics, total anthocyanins, and phenolic acids, despite some modifications being observed in the metabolomics profile of the berries. Yet, additional studies on other grapevine cultivars, with different drought tolerance, and DDR sub-regions, complemented with soil analysis (i.e., granulometry, soil water potential) berry and wine metabolomics, and wine tasting approaches would strengthen the present data. Besides the scientific relevance of these studies, results may aid viticulturists and decision makers to implement and optimize irrigation in Douro region.

## Data Availability

The following are available in Supplementary Materials.

## Supplementary Materials

The following supporting information can be downloaded at: https://www.mdpi.com/article/10.3390/plants11060732/s1, Figure S1: Monthly mean climatic conditions that occurred during 2018 vintage: Figure S2: Monthly mean climatic conditions that occurred during 2019; Figure S3: Monthly mean climatic conditions that occurred during 2020; Table S1: Berry quality attributes during maturation until harvest (eight weeks after veraison—WAV) in 2018; Table S2: Berry quality attributes during maturation until harvest (from four to eight week after veraison—WAV) in the 2018 vintage. Table S3: Berry quality attributes during maturation until harvest (seven weeks after veraison—WAV) in the 2019 vintage. Table S4: Berry quality attributes during maturation until harvest (from two to seven weeks after veraison—WAV), in the 2019 vintage. Table S5: Berry quality attributes during maturation until harvest (seven weeks after veraison—WAV) in the 2020 vintage. Table S6: Berry quality attributes during maturation until harvest (from two to seven weeks after veraison—WAV), in the 2020 vintage. Table S7: Metabolites identified by UPLC–MS in mature grape berries of Touriga Nacional in 2018, 2019, and 2020 vintages.
