# Supplementary material for: Impact of Deficit Irrigation on Grapevine cv. ‘Touriga Nacional’ during Three Seasons in Douro Region: An Agronomical and Metabolomics Approach
Source: Plants (Basel). 2022 Mar 9;11(6):732. doi: 10.3390/plants11060732 (PMC8956047; doi:10.3390/plants11060732)
Supplement: Supplementary file 1 [file plants-11-00732-s001.zip › plants-1583848_SUP.pdf]

plants

Impact of Deficit Irrigation on Grapevine cv. 'Touriga Nacional' during Three Seasons in Douro Region: An Agronomical and Metabolomics Approach

Inês L. Cabral [1], António Teixeira [2], Arnaud Lanoue [3], Marianne Unlubayir [3], Thibaut Munsch [3], Joana Valente [4], Fernando Alves [4], Pedro Leal da Costa [4], Frank S. Rogerson [4], Susana M. P. Carvalho [1], Hernâni Gerós [2,*] and Jorge Queiroz [1]

[1] GreenUPorto—Research Centre on Sustainable Agrifood Production / Inov4Agro & DGAOT, Faculty of Sciences, University of Porto, Campus de Vairão, Rua da Agrária 747, 4485-646 Vairão, Portugal; ines.cabral@fc.up.pt (I.L.C); susana.carvalho@fc.up.pt (S.M.P.C); jqueiroz@fc.up.pt (J.Q)

[2] Centre of Molecular and Environmental Biology (CBMA), Department of Biology, Campus de Gualtar, University do Minho, 4710-057 Braga, Portugal; antonio.teixeira@bio.uminho.pt

[3] EA2106 Biomolécules et Biotechnologies Végétales, Université de Tours, 37200 Tours, France; arnaud.lanoue@univ-tours.fr (A.L.); marianne.unlubayir@univ-tours.fr (M.U.); thibaut.munsch@univ-tours.fr (T.M.)

[4] Symington Family Estates, Vinhos SA, Travessa Barão de Forrester 86, 4431-901 Vila Nova de Gaia, Portugal; joana.valente@symington.com (J.V.); fernando.alves@symington.com (F.A.); pedro.leal.costa@symington.com (P.L.d.C); frank.rogerson@symington.com (F.S.R.)

* Correspondence: geros@bio.uminho.pt; Tel.: (+351 253 605 812)

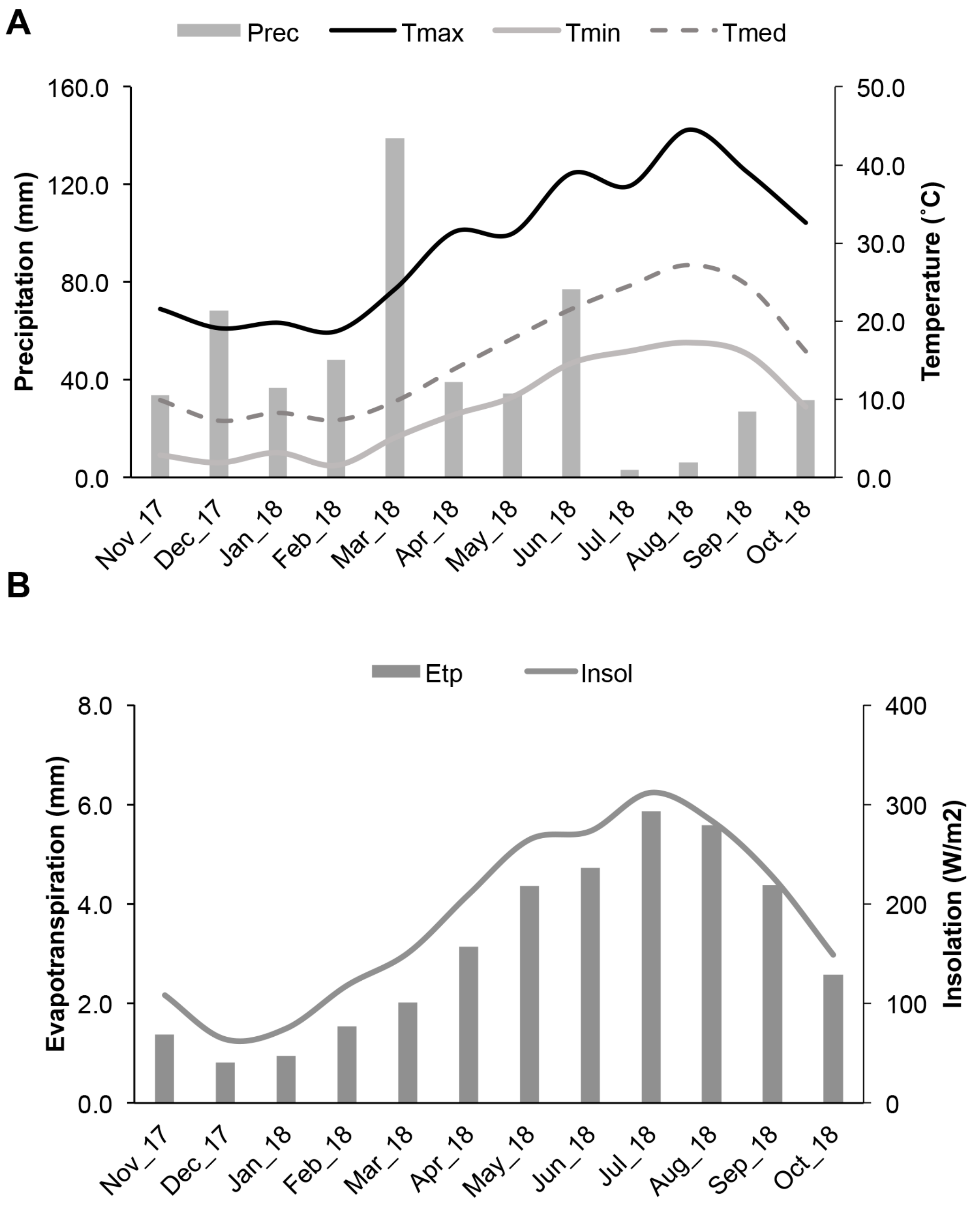

Figure S1. Monthly mean climatic conditions occurred during the 2018 vintage in the vineyard Quinta do Ataíde from the Douro Superior sub-region. **A**) Precipitation-Prec. (mm); Maximum temperature -Tmax-(°C); Minimum temperature – Tmin (°C) and Mean temperature – Tavg (°C). **B**) Evapotranspiration - Etp (mm); Insolation – Insol. (W/m^2). Arrows indicate the occurrence of heat waves.

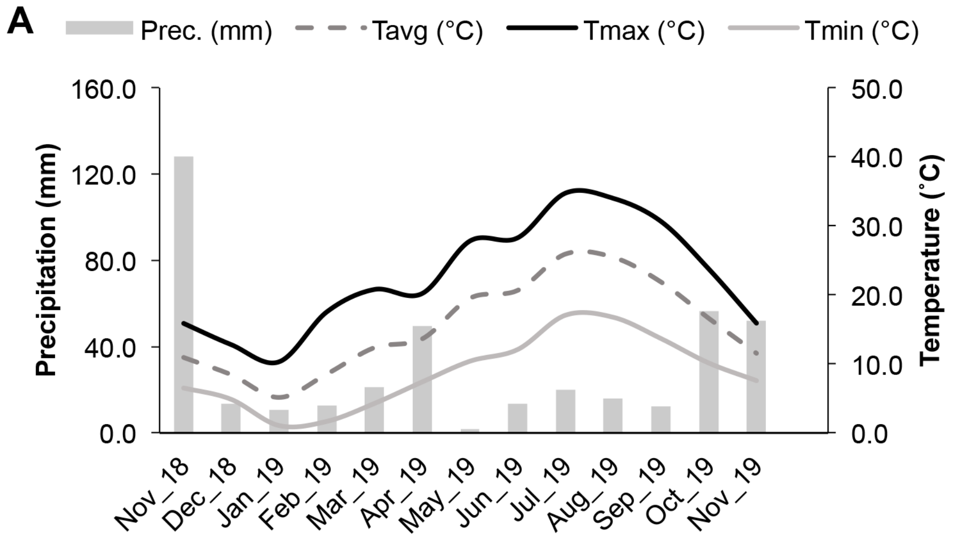

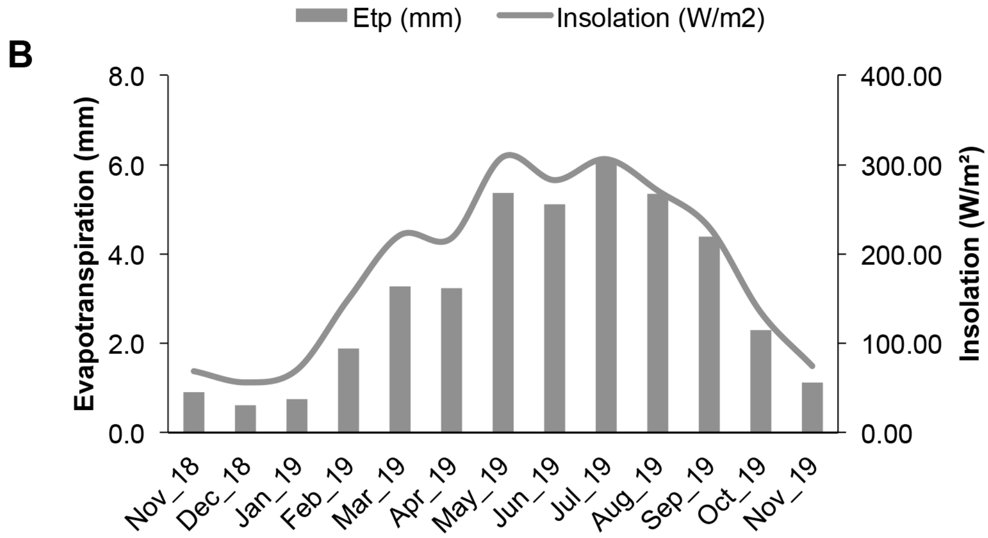

Figure S2. Monthly mean climatic conditions occurred during the 2019 vintage in the vineyard Quinta do Ataíde from the Douro Superior sub-region. **A**) Precipitation-Prec. (mm); Maximum temperature -Tmax-(°C); Minimum temperature – Tmin (°C) and Mean temperature – Tavg (°C). **B**) Evapotranspiration - Etp (mm); Insolation – Insol. (W/m^2).

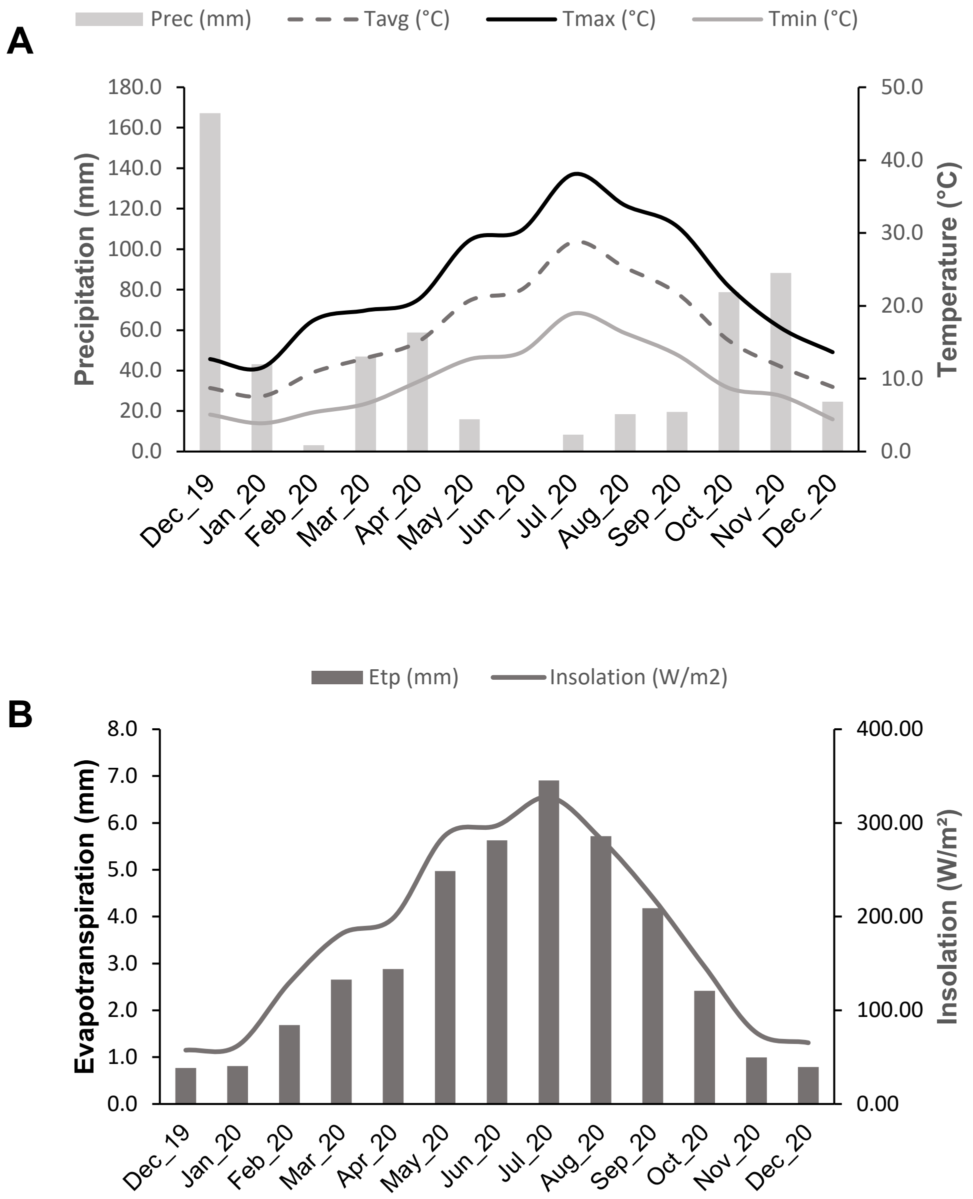

Figure S3. Monthly mean climatic conditions occurred during the 2020 vintage in the vineyard Quinta do Ataíde from the Douro Superior sub-region. A) Precipitation-Prec. (mm); Maximum temperature -Tmax-(°C); Minimum temperature – Tmin (°C) and Average temperature – Tavg (°C). B) Evapotranspiration - Etp (mm); Insolation – Insol. (W/m^2).

Table S1. Berry quality attributes during maturation until harvest (eight weeks after *veraison* - WAV) in **2018** vintage. Irrigation conditions: R0 = non-irrigated plants; R30 = deficit irrigation corresponding to 30% of evapotranspiration; R70 = deficit irrigation corresponding to 70% of evapotranspiration. [1] Sig. indicate significant differences between varieties: "n.s." – not significant; "*" – p < 0.05; "**" – p < 0.01.

Berry quality	Treatment	WAV 4	WAV 5	WAV 6	WAV 7	WAV 8
Berry weight (g)	R0	1.055 b	1.061 b	1.109 b	1.148 b	1.093
	R30	1.341 ab	1.433 a	1.416 a	1.506 a	1.311
	R70	1.505 a	1.479 a	1.571 a	1.507 a	1.390
	Sig.[1]	**	**	**	*	n.s.
pH	R0	3.325	3.625	3.470	4.410	3.725
	R30	3.330	3.565	3.483	3.725	3.740
	R70	3.413	3.608	3.538	3.760	3.795
	Sig.[1]	n.s.	n.s.	n.s.	n.s.	n.s.
TSS (°Brix)	R0	18.662	19.094	20.295	21.294	22.622 b
	R30	19.116	19.764	20.840	22.316	23.927 ab
	R70	21.137	21.492	22.194	23.387	25.205 a
	Sig.[1]	n.s.	n.s.	n.s.	n.s.	*
Total Acidity	R0	6.558 a	4.775	5.645	4.510	4.283
	R30	6.280 ab	4.695	5.418	4.143	4.163
	R70	5.453 b	4.440	4.780	3.965	3.998
	Sig.[1]	*	n.s.	n.s.	n.s.	n.s.
Malic Acid	R0	1.600	1.075	0.875	1.050	0.975
	R30	1.525	1.025	0.875	0.950	0.975
	R70	1.200	0.900	0.625	0.850	0.775
	Sig.[1]	n.s.	n.s.	n.s.	n.s.	n.s.
YAN	R0	288.125	251.300	268.200	234.975	271.500
	R30	258.750	192.025	224.225	203.375	222.225
	R70	202.000	187.525	194.725	161.775	204.400
	Sig.[1]	n.s.	n.s.	n.s.	n.s.	n.s.
Total Phenols	R0	11.703	16.473	14.683	14.788	17.628
	R30	8.824	12.591	11.183	12.874	13.551
	R70	8.391	11.169	10.619	13.894	13.191
	Sig.[1]	n.s.	n.s.	n.s.	n.s.	n.s.

Table S2. Berry quality attributes during maturation until harvest (from four to eight week after *veraison* - WAV) in **2018** vintage. Irrigation conditions: R0 = non-irrigated plants; R30 = deficit irrigation corresponding to 30% of evapotranspiration; R70 = deficit irrigation corresponding to 70% of evapotranspiration.

Berry quality	R0A	R0B	R0C	R0D	R30A	R30B	R30C	R30D	R70A	R70B	R70C	R70D
Berry weight 4	0.926	0.948	0.904	1.44	1.234	1.35	1.354	1.426	1.462	1.468	1.552	1.538
Berry weight 5	1.014	0.91	0.908	1.41	1.306	1.43	1.518	1.478	1.51	1.368	1.606	1.43
Berry weight 6	1.066	0.946	1.004	1.42	1.312	1.402	1.528	1.422	1.478	1.562	1.594	1.648
Berry weight 7	0.952	1.14	1.002	1.498	1.628	1.482	1.522	1.39	1.454	1.468	1.526	1.578
Berry weight 8	0.948	1.008	1.034	1.38	1.222	1.182	1.492	1.346	1.406	1.236	1.496	1.422
pH 4	3.3	3.3	3.23	3.47	3.28	3.28	3.33	3.43	3.34	3.4	3.58	3.33
pH 5	3.59	3.62	3.65	3.64	3.54	3.42	3.72	3.58	3.54	3.52	3.76	3.61
pH 6	3.42	3.43	3.44	3.59	3.43	3.47	3.5	3.53	3.45	3.45	3.77	3.48
pH 7	3.6	3.64	3.66	3.74	3.77	3.64	3.73	3.76	3.74	3.69	3.96	3.65
pH 8	3.65	3.82	3.69	3.74	3.71	3.68	3.77	3.8	3.72	3.77	3.96	3.73
TSS 4	18.378	19.044	18.216	19.008	18.594	18.702	19.404	19.764	19.098	21.024	24.894	19.53
TSS 5	18.684	18.9	19.206	19.584	18.864	19.71	20.142	20.34	19.638	21.672	24.426	20.232
TSS 6	20.142	20.124	20.178	20.736	20.484	20.97	20.754	21.15	21.024	21.438	25.686	20.628
TSS 7	21.276	21.096	21.096	21.708	21.708	22.248	22.572	22.734	22.05	22.716	26.334	22.446
TSS 8	22.662	22.698	21.87	23.256	23.076	23.688	24.516	24.426	24.102	25.074	27.846	23.796
Total acidity 4	6.69	6.46	7.12	5.96	6.57	6.26	6.03	6.26	5.53	5.7	4.62	5.96
Total acidity 5	4.9	4.76	4.82	4.62	4.71	4.46	4.7	4.91	4.46	4.98	3.88	4.44
Total acidity 6	5.89	5.75	5.89	5.05	5.5	5.05	5.12	6	5.02	5.13	3.8	5.17
Total acidity 7	4.83	4.47	4.44	4.3	4.02	4.25	4.12	4.18	3.86	4.24	3.32	4.44
Total acidity 8	4.63	3.94	4.22	4.34	4.22	4.2	4.07	4.16	4.15	4.21	3.33	4.3
Malic acid 4	1.6	1.2	1.5	2.1	1.4	1.2	1.4	2.1	1.2	1.4	0.8	1.4
Malic acid 5	1.2	0.7	1	1.4	0.9	0.7	1.1	1.4	0.8	1.1	0.6	1.1
Malic acid 6	0.7	0.7	0.8	1.3	0.7	0.5	0.8	1.5	0.6	0.8	0.2	0.9
Malic acid 7	0.9	0.8	1	1.5	1	0.6	0.9	1.3	0.8	0.9	0.7	1
Malic acid 8	0.8	0.8	1.1	1.2	0.9	0.5	0.9	1.6	0.7	0.8	0.7	0.9
YAN 4	259.7	252.7	288	352.1	245.6	187.1	235.4	366.9	262	148.1	131.4	266.5
YAN 5	276.9	199.7	270.2	258.4	228.2	112.2	174.3	253.4	228.2	147.8	137.2	236.9
YAN 6	222.4	236.6	279.4	334.4	248.1	173.7	232.1	243	271.9	133.7	150.7	222.6
YAN 7	207.4	196.5	228.8	307.2	204.1	158.3	212.7	238.4	227.3	93.6	95.8	230.4
YAN 8	236.1	226.7	264.9	358.3	225.3	137.6	239.4	286.6	288.5	133.8	131.6	263.7
Total phenols 4	11.398	14.358	13.378	7.679	9.649	8.549	7.669	9.429	6.409	8.579	11.648	6.929

**Berry quality**	**R0A**	**R0B**	**R0C**	**R0D**	**R30A**	**R30B**	**R30C**	**R30D**	**R70A**	**R70B**	**R70C**	**R70D**
Total phenols 5	15.848	21.047	19.548	9.449	11.618	14.328	15.088	9.329	9.539	11.868	13.718	9.549
Total phenols 6	14.338	18.328	15.388	10.679	11.788	11.968	11.898	9.079	8.949	10.769	13.378	9.379
Total phenols 7	14.824	17.463	15.793	11.069	14.954	14.074	13.449	9.024	11.294	13.894	18.708	11.684
Total phenols 8	17.548	23.957	16.908	12.098	14.148	14.848	14.128	11.078	10.879	14.758	15.598	11.528
Number of Shoots	17.250	13.875	14.375	16.625	13.875	14.875	15.75	17.000	17.000	16.625	16.125	16.000
Number of clusters 8	28.500	21.000	15.000	21.500	31.000	25.000	19.000	25.000	32.500	22.000	11.500	22.000
Pruning Weight	0.456	0.368	0.466	0.533	0.466	0.404	0.504	0.653	0.359	0.346	0.254	0.448
Clusters weight 8	1.395	1.25	1.2688	1.7825	2.0975	2.0063	1.8763	1.6688	2.1075	2.0775	0.6625	2.415
Foliar Area	3.057	1.002	4.08	1.86	3.178	9.164	2.85	3.109	1.132	3.787	3.493	1.96

Table S3. Berry quality attributes during maturation until harvest (seven weeks after *veraison* - WAV) in **2019** vintage. Irrigation conditions: R0 = non-irrigated plants; R30 = deficit irrigation corresponding to 30% of evapotranspiration; R70 = deficit irrigation corresponding to 70% of evapotranspiration. [1] Sig. indicate significant differences between varieties: "n.s." – not significant; "*" – $p < 0.05$; "**" – $p < 0.01$; "***" - $p < 0.001$; "****" – $p < 0.0001$.

Berry quality	Treatment	WAV 2	WAV 4	WAV 6	WAV 7
**Berry weight (g)**	R0	1.151 b	1.192 b	1.282 b	1.287 b
	R30	1.596 a	1.649 a	1.583 a	1.531 a
	R70	1.583 a	1.656 a	1.657 a	1.581 a
	Sig.[1]	****	****	***	***
**pH**	R0	3.228	3.460	3.640	3.763
	R30	3.273	3.525	3.690	3.833
	R70	3.255	3.523	3.680	3.758
	Sig.[1]	n.s.	n.s.	n.s.	n.s.
**TSS (°Brix)**	R0	18.80	22.06	23.45	23.33
	R30	19.53	22.98	23.95	23.72
	R70	19.91	23.24	23.79	23.83
	Sig.[1]	n.s.	n.s.	n.s.	n.s.
**Total Acidity**	R0	7.15	5.08	4.26	3.62
	R30	7.43	5.06	4.50	3.68
	R70	7.72	5.26	4.58	4.03
	Sig.[1]	n.s.	n.s.	n.s.	n.s.
**Malic Acid**	R0	2.03	1.05	1.00	0.63 b
	R30	2.70	1.55	1.33	1.08 a
	R70	2.88	1.57	1.43	1.03 a
	Sig.[1]	n.s.	n.s.	n.s.	*
**YAN**	R0	181.20	210.90	194.50	215.70
	R30	222.40	216.30	203.00	201.40
	R70	218.60	256.60	204.51	180.20
	Sig.[1]	n.s.	n.s.	n.s.	n.s.
**Total Phenols**	R0	11.35	12.76	14.21	15.26
	R30	8.83	12.69	12.35	14.46
	R70	9.33	11.55	13.02	12.21
	Sig.[1]	n.s.	n.s.	n.s.	n.s.

Table S4. Berry quality attributes during maturation until harvest (from two to seven weeks after *veraison* - WAV), in **2019** vintage. Irrigation conditions: R0 = non-irrigated plants; R30 = deficit irrigation corresponding to 30% of evapotranspiration; R70 = deficit irrigation corresponding to 70% of evapotranspiration.

Berry quality	R0A	R0B	R0C	R0D	R30A	R30B	R30C	R30D	R70A	R70B	R70C	R70D
Berry weight 2	1.19	1.07	1.12	1.22	1.48	1.67	1.65	1.58	1.67	1.50	1.55	1.61
Berry weight 4	1.21	1.18	1.17	1.21	1.60	1.63	1.71	1.65	1.72	1.61	1.65	1.64
Berry weight 6	1.32	1.22	1.26	1.33	1.52	1.54	1.65	1.62	1.70	1.53	1.63	1.76
Berry weight 7	1.28	1.26	1.20	1.40	1.51	1.44	1.61	1.57	1.63	1.53	1.54	1.63
pH 2	3.28	3.18	3.12	3.33	3.31	3.35	3.17	3.26	3.25	3.19	3.33	3.25
pH 4	3.51	3.46	3.40	3.47	3.59	3.56	3.49	3.46	3.55	3.53	3.51	3.50
pH 6	3.69	3.71	3.40	3.76	3.75	3.73	3.66	3.62	3.71	3.71	3.67	3.63
pH 7	3.78	3.78	3.70	3.79	3.86	3.88	3.84	3.75	3.80	3.78	3.73	3.72
TSS 2	19.71	19.49	18.04	17.96	20.39	20.64	18.95	18.13	19.28	20.16	20.86	19.35
TSS 4	22.55	23.00	21.29	21.40	23.72	23.83	22.46	21.89	22.41	23.99	24.08	22.48
TSS 6	23.76	24.03	23.04	22.97	24.733	24.79	23.54	22.75	23.31	24.14	24.41	23.31
TSS 7	24.12	23.832	23.004	22.356	23.922	24.84	23.814	22.32	22.644	25.02	24.282	23.31
Total acidity 2	6.74	6.91	7.92	7.04	7.1	6.46	8.02	8.14	7.51	8.51	7.18	7.68
Total acidity 4	4.88	4.95	5.44	5.06	4.72	4.77	5.19	5.54	5.07	5.48	5.2	5.29
Total acidity 6	4.25	4.17	4.54	4.08	4.38	4.28	4.64	4.69	4.32	4.72	4.55	4.74
Total acidity 7	3.48	3.4	4	3.61	3.52	3.44	3.55	4.19	3.74	3.96	4.01	4.41
Malic acid 2	1.9	1.5	2.2	2.5	2.4	2	2.7	3.7	2.7	3.2	2.7	2.9
Malic acid 4	1.2	0.7	1.2	1.1	1.4	1.1	1.6	2.1	1.7	1.7	1.5	1.4
Malic acid 6	0.9	0.8	1	1.3	1.2	1	1.3	1.8	1.6	1.6	1.1	1.4
Malic acid 7	0.6	0.6	0.6	0.7	1	0.8	1.1	1.4	1.2	0.9	0.8	1.2
YAN 2	184.7	133.3	160.6	246.1	246.2	165	167.9	310.5	255.8	200.4	163.4	254.9
YAN 4	233.2	183.2	164.8	262.2	226.8	135.2	209.8	293.5	270.5	274.4	197.3	284.1
YAN 6	189	174.5	163.9	250.6	206.5	155.7	185	264.9	230.5	184.8	159.7	242.8
YAN 7	180.6	191.6	213.5	277	208.4	116.7	208.8	271.5	211.1	176.8	142.5	190.3
Total phenols 2	11.832	10.499	10.932	12.118	9.319	10.259	8.152	7.586	6.772	9.632	12.078	8.832
Total phenols 4	11.758	14.165	13.738	11.372	14.738	13.885	12.225	9.912	9.819	11.872	13.072	11.445
Total phenols 6	12.825	16.711	13.552	13.765	12.312	13.905	13.078	10.092	11.658	15.118	13.498	11.818
Total phenols 7	12.932	16.351	19.898	11.872	13.938	19.038	14.785	10.079	11.592	11.398	12.458	13.398
Number of Shoots	11.75	10.125	11.375	11.125	9.000	9.625	10.25	12.375	10.75	11.75	11.375	11.875
Number of clusters 7	16.25	16.125	16.75	17.375	12.125	18.25	17.75	24.00	13.25	16.375	13.25	15.875
Pruning Weight	0.139	0.161	0.160	0.155	0.177	0.276	0.245	0.478	0.175	0.311	0.227	0.263
Clusters weight 7	0.713	0.903	0.989	1.239	0.923	1.455	1.666	2.429	1.118	1.204	0.845	1.434
Foliar Area	1.883	1.887	2.769	1.635	3.122	3.290	3.850	4.798	2.046	4.827	2.465	3.783

Table S5. Berry quality attributes during maturation until harvest (seven weeks after *veraison* - WAV) in **2020** vintage. Irrigation conditions: R0 = non-irrigated plants; R30 = deficit irrigation corresponding to 30% of evapotranspiration; R70 = deficit irrigation corresponding to 70% of evapotranspiration. [1] Sig. indicate significant differences between varieties: "n.s." – not significant; "*" – $p < 0.05$; "**" – $p < 0.01$; "***" - $p < 0.001$; "****" – $p < 0.0001$.

Berry quality	Treatment	WAV 5	WAV 6	WAV 7
Berry weight (g)	R0	1.30 b	1.22 a	1.23
	R30	1.68 a	1.65 ab	1.56
	R70	1.69 a	1.76 b	1.53
	Sig.[1]	*	*	n.s.
pH	R0	3.63	3.73	3.81
	R30	3.67	3.78	3.82
	R70	3.74	3.75	3.84
	Sig.[1]	n.s.	n.s.	n.s.
TSS (°Brix)	R0	19.93	21.55	22.38
	R30	20.11	21.87	22.78
	R70	21.89	22.20	24.26
	Sig.[1]	n.s.	n.s.	n.s.
Total Acidity	R0	4.49	4.12	3.97
	R30	4.30	3.89	3.81
	R70	4.18	3.92	3.91
	Sig.[1]	n.s.	n.s.	n.s.
Malic Acid	R0	1.23	0.75	1.05
	R30	1.28	0.73	1.08
	R70	1.48	0.70	1.10
	Sig.[1]	n.s.	n.s.	n.s.
YAN	R0	248.80	252.80	253.80
	R30	189.30	140.90	175.60
	R70	215.10	175.70	198.60
	Sig.[1]	n.s.	n.s.	n.s.
Total Phenols	R0	14.36	14.04 a	14.30
	R30	11.22	11.09 ab	
	R70	12.10	9.41 b	
	Sig.[1]	n.s.	*	n.s.

Table S6. Berry quality attributes during maturation until harvest (from two to seven weeks after *veraison* - WAV), in **2020** vintage. Irrigation conditions: R0 = non-irrigated plants; R30 = deficit irrigation corresponding to 30% of evapotranspiration; R70 = deficit irrigation corresponding to 70% of evapotranspiration.

**Berry quality**	**R0A**	**R0B**	**R0C**	**R0D**	**R30A**	**R30B**	**R30C**	**R30D**	**R70A**	**R70B**	**R70C**	**R70D**
Berry weight 5	1.26	1.07	1.26	1.60	1.68	1.85	1.60	1.57	1.72	1.65	1.49	1.88
Berry weight 6	1.31	1.02	0.98	1.55	1.74	1.74	1.53	1.60	1.72	1.84	1.39	2.07
Berry weight 7	1.21	1.00	1.22	1.53	1.61	1.58	1.68	1.38	1.55	1.50	1.26	1.82
pH 5	3.76	3.50	3.57	3.67	3.80	3.68	3.58	3.60	3.67	3.79	3.72	3.77
pH 6	3.91	3.61	3.70	3.70	3.83	4.00	3.61	3.69	3.77	3.79	3.69	3.75
pH 7	3.95	3.63	3.78	3.88	3.92	3.93	3.78	3.65	3.85	3.91	3.74	3.84
TSS 5	20.52	19.30	19.60	20.29	21.42	21.49	19.71	17.80	19.80	21.92	24.55	21.29
TSS 6	22.03	21.20	21.83	21.11	22.50	23.40	21.83	19.76	21.44	22.05	24.01	21.31
TSS 7	22.66	21.58	22.46	22.80	23.89	24.08	22.39	20.75	22.66	24.64	26.28	23.45
Total acidity 5	4.18	4.64	4.47	4.67	3.95	4.03	4.37	4.85	4.31	3.95	4.15	4.31
Total acidity 6	3.77	4.24	4.20	4.27	3.72	3.50	4.04	4.31	3.96	3.81	3.99	3.90
Total acidity 7	3.73	4.32	4.05	3.79	3.50	3.57	3.66	4.49	3.79	3.81	4.13	3.89
Malic acid 5	1.50	0.80	1.10	1.50	1.30	1.10	1.00	1.70	1.50	1.40	1.10	1.90
Malic acid 6	1.10	0.20	0.80	0.90	0.80	0.80	0.30	1.00	1.00	0.60	0.30	0.90
Malic acid 7	1.30	0.70	0.90	1.30	1.10	1.00	0.80	1.40	1.30	1.10	0.60	1.40
YAN 5	285.90	182.70	179.50	347.00	200.70	120.00	136.70	299.60	239.70	202.30	107.80	310.60
YAN 6	320.40	145.30	236.30	309.10	129.50	95.10	72.20	266.70	227.40	162.70	75.20	237.50
YAN 7	309.00	219.00	183.60	303.60	163.00	127.00	150.20	262.20	218.20	180.00	105.10	290.90
Total phenols 5	15.31	15.81	13.35	12.96	12.40	10.36	13.98	8.13	3.66	12.96	16.15	9.62
Total phenols 6	14.38	14.58	16.06	11.15	9.53	13.90	12.16	8.76	7.99	9.63	12.04	8.00
Total phenols 7	14.75	15.70	14.84	11.90	11.39	13.90	12.95	8.54	10.71	11.41	13.86	9.41
Number of Shoots	16.50	13.13	13.63	14.13	13.25	14.25	14.25	14.50	16.50	17.63	14.88	16.00
Number of clusters 7	17.25	17.50	18.88	18.25	16.63	22.13	21.50	32.75	20.88	26.88	20.00	28.00
Pruning Weight	0.55	0.41	0.42	0.59	0.63	0.60	0.57	0.77	0.71	0.64	0.42	0.72
Clusters weight 7	1.01	1.30	1.32	1.88	1.58	1.82	2.18	3.95	2.60	1.90	1.31	2.53
Foliar Area	2.64	1.24	3.09	4.45	3.29	3.16	1.77	1.46	1.87	4.03	2.80	3.55

Table S7. Metabolites identified by UPLC-MS in mature grape berries of Touriga Nacional in 2018, 2019 and 2020 vintages from Douro region subjected to different irrigation conditions: R0 = non-irrigated plants; R30 = deficit irrigation corresponding to 30% of evapotranspiration; R70 = deficit irrigation corresponding to 70% of evapotranspiration. Values represent the mean ± SD (*n* = 4) of arbitrary units normalized by the weight of lyophilized grape berry powder.

			R0						R30						R70					
code	**Compound**	Group	2018		2019		2020		2018		2019		2020		2018		2019		2020	
			mean	sd	mean	sd	mean	sd	mean	sd	mean	sd	mean	sd	mean	sd	mean	sd	mean	sd
m0	L-proline	Amino acids	354429.7	37658.7	426780.4	59804.7	408503.2	51685.8	373649.9	87044.4	484751.3	77170.5	439951.3	7110.9	423435.6	3818.7	446189.1	69251.6	485141.9	14679.3
m1	L-leucine	Amino acids	119469.5	214.3	29777.1	2132.4	64394.6	3216.9	70631.7	3974.5	35646.7	8470.7	42415.2	5727.5	35847.3	2276.7	24665.6	3300.6	39020.4	3676.2
m2	L-isoleucine	Amino acids	162392.4	31792.4	50441.6	2478.8	121674.9	13095.7	121737.7	3118.3	59408.7	5076.0	80528.4	10297.4	68825.2	713.9	50590.2	6252.7	78407.0	7037.0
m3	phenylalanine	Amino acids	31960.4	4427.8	19287.8	803.1	37070.9	5221.5	33409.0	1943.5	25904.1	2417.4	27222.3	3102.1	20983.6	580.1	24355.4	2069.9	29364.2	3538.9
m4	L-tyrosine	Amino acids	33021.1	2239.1	13486.3	1540.1	31023.0	3349.5	32367.2	1302.3	22624.6	1861.8	30640.8	4418.8	20758.4	1140.3	17096.0	3003.4	30877.5	2937.3
m5	L-tryptophan	Amino acids	145623.4	25038.5	59351.5	4839.7	105010.3	8523.2	102685.9	5321.9	60133.9	6590.0	74746.1	10565.7	85769.1	9914.6	46011.9	6178.1	67153.4	5842.6
m6	cyanidin-3-O-glucoside	Anthocyanin di-OH	3972.7	664.8	12051.8	158.8	4750.0	512.4	13208.2	1092.3	11633.8	2232.0	7012.1	491.7	10106.1	902.8	13498.6	4346.7	7795.9	597.8
m7	peonidin-3-O-glucoside	Anthocyanin di-OH	37247.2	4307.3	76706.3	12490.9	37550.1	3972.7	102507.1	15199.5	79290.5	14634.1	48590.6	1503.7	72981.4	8159.1	83923.6	7316.2	49252.0	4947.9
m8	cyanidin-3-O-(6-O-acetyl)-glucoside	Anthocyanin di-OH	556.5	42.0	1455.9	325.2	916.7	91.5	1776.3	68.2	1677.3	91.9	1203.4	59.9	1269.9	55.9	1825.9	444.0	1196.3	161.6
m9	delphinidin-3-O-glucoside	Anthocyanin tri-OH	24804.2	199.2	39870.0	5013.3	20987.4	1940.1	28508.8	2010.0	49804.2	3128.0	27339.8	1358.6	27601.2	2801.6	67070.7	20498.5	34867.6	2717.3
m10	petunidin-3-O-glucoside	Anthocyanin tri-OH	41557.9	10860.0	65379.9	10496.3	50115.5	7046.1	49955.9	6126.2	74799.3	3205.1	48686.6	1527.5	48640.3	2449.4	96514.3	20719.9	59763.8	3302.7
m11	malvidin-3-O-glucoside	Anthocyanin tri-OH	426102.1	19556.4	352206.1	41952.3	1151.9	114.9	309214.0	48014.8	324584.7	43840.3	318935.4	9985.4	327110.4	34355.3	347633.6	10567.3	314439.1	12844.6
m12	petunidin-3-O-(6-O-acetyl)-glucoside	Anthocyanin tri-OH	10557.1	1376.3	18397.1	2398.2	314596.2	43547.9	11665.1	1844.1	20372.9	2912.4	13338.4	448.8	10281.0	776.5	28719.1	6127.9	16609.2	1029.1
m13	malvidin-3-O-(6-O-acetyl)-glucoside	Anthocyanin tri-OH	309449.3	44547.5	225337.7	33026.5	11661.1	1406.2	248972.0	31674.6	228876.6	24980.2	238540.6	12743.3	245240.0	17988.0	209890.9	21465.4	236781.2	11232.9
m14	petunidin-3-O-(6-p-coumaroyl)-glucoside	Anthocyanin tri-OH	22026.9	279.6	18238.1	1609.9	297292.7	30076.2	19353.4	2868.5	18810.4	2622.4	22586.9	2793.7	18710.5	1792.2	20443.3	2023.4	19674.8	2614.6
m15	malvidin-3-O-(6-p-coumaroyl)-glucoside	Anthocyanin tri-OH	258693.5	37277.6	171735.0	25631.5	16275.3	1324.5	208048.6	20432.2	171328.0	22214.7	208644.0	22454.2	216192.3	3827.5	148705.8	28805.6	186992.4	6335.5
m16	malvidin-3,5-O-diglucoside	Anthocyanin tri-OH	1734.2	215.3	1683.6	233.8	215193.7	19089.1	2142.4	263.6	1069.2	253.0	1568.3	136.0	1783.4	220.9	1048.2	148.0	1552.8	88.4
m17	catechin	Flavan-3-ols	2099.6	697.3	1997.0	292.9	1053.9	138.8	1736.1	134.0	2646.7	319.1	1238.1	136.7	2236.2	196.3	2928.6	89.9	1930.4	247.4
m18	epicatechin	Flavan-3-ols	2254.9	96.3	1576.8	318.8	953.1	59.9	2078.3	256.7	3186.5	468.7	966.9	118.3	3094.1	204.4	2502.9	433.4	1691.5	352.3

m19	catechin gallate	Flavan-3-ols	81.3	na	197.5	46.8	191.8	24.6	190.4	14.2	264.8	52.2	186.9	34.9	70.2	7.9	577.2	58.6	198.9	20.2
m20	procyanidinB1	Flavan-3-ols	3400.8	241.3	2721.8	318.2	3148.9	188.3	2681.4	194.6	2488.7	300.8	2603.3	484.0	2544.1	132.1	2918.9	329.3	2840.9	302.7
m21	procyandinB2	Flavan-3-ols	4935.5	136.2	3637.9	503.2	3053.5	420.6	3709.4	176.9	4297.5	756.6	2827.0	361.0	3721.6	280.6	4001.4	503.4	3582.2	379.9
m22	procyanidinB3	Flavan-3-ols	937.5	68.6	1133.8	132.4	624.2	120.4	852.2	30.7	1414.6	176.0	829.7	135.7	946.7	41.6	1367.9	81.0	971.7	83.4
m23	procyanidinB4	Flavan-3-ols	1862.9	276.6	1387.9	226.7	963.6	59.7	1422.5	148.4	1942.3	433.6	862.5	133.4	1478.7	74.9	1665.0	201.8	1057.1	164.0
m24	procyanidin gallate 1	Flavan-3-ols	368.9	55.1	709.4	2.9	792.6	128.5	571.3	93.4	883.0	93.5	893.5	149.1	641.8	13.3	687.6	155.7	712.4	79.6
m25	procyanidin trimer 2	Flavan-3-ols	1605.3	233.3	1219.4	112.5	1296.0	69.6	1271.6	177.7	1487.5	176.9	1125.2	192.6	1584.7	118.5	1618.8	165.0	1179.6	32.4
m26	procyanidin gallate 2	Flavan-3-ols	898.4	6.6	709.4	117.0	691.5	75.3	566.2	57.1	736.4	81.3	647.7	93.0	718.5	34.8	809.4	108.1	617.3	18.8
m27	procyanidin trimer1	Flavan-3-ols	988.4	34.8	644.5	44.6	838.1	179.0	729.4	47.8	852.2	115.5	603.7	103.4	696.3	36.0	895.5	90.0	753.9	81.7
m28	kaempferol-3-O-glucoside	Flavonols	425.8	71.3	456.7	50.6	68.2	7.8	301.7	47.3	564.9	157.6	484.7	47.2	150.7	24.4	668.3	153.5	365.1	16.5
m29	quercetin-3-O-glucoside	Flavonols	1322.4	101.8	2528.6	224.8	1480.6	167.0	1905.5	192.8	2816.1	575.8	2018.0	299.3	1524.9	257.3	3860.9	110.4	1806.8	146.9
m30	quercetin-3-O-glucuronide	Flavonols	2420.4	419.1	4339.7	287.7	1712.4	196.6	3170.7	507.2	4002.0	532.5	2221.3	171.5	3480.2	573.9	4816.3	1561.9	2060.7	176.6
m31	myricetin-hexoside1	Flavonols	527.8	51.9	1202.6	195.5	488.3	31.2	1737.6	250.2	1074.2	134.5	762.9	68.6	1122.9	70.8	1218.4	165.4	800.0	42.7
m32	myricetin glucoside	Flavonols	5564.2	717.9	5002.7	280.4	5030.4	522.2	4233.4	599.0	5247.3	683.3	4360.7	503.0	3374.4	500.0	6087.4	320.8	4315.0	123.9
m33	quercetin derivative	Flavonols	774.3	45.8	1115.5	212.4	822.7	169.6	842.1	94.2	1256.8	112.2	809.8	43.2	851.3	83.3	1642.3	414.8	929.2	103.7
m34	kaempferol-3-O-rutinoside	Flavonols	71.4	na	88.8	7.6	133.9	9.0	170.2	7.4	114.9	3.0	90.6	17.2	150.4	14.5	116.3	30.0	117.6	19.2
m35	gallic acid	Phenolic acids	415.0	48.0	194.3	71.3	222.0	44.1	303.6	36.0	513.2	29.9	100.2	14.7	262.8	17.8	354.8	23.7	111.0	10.9
m36	citric acid	Phenolic acids	10409.1	310.0	8489.1	256.0	11939.4	752.3	10227.5	431.8	9948.4	980.7	10761.7	1437.5	9139.6	1218.9	12354.0	1273.2	10327.6	1124.2
m37	coutaric acid	Phenolic acids	9414.7	5.4	6669.4	297.6	4841.0	484.1	7560.0	474.8	6201.4	918.1	5402.0	338.6	6834.3	958.5	7921.3	161.3	5218.1	148.6
m38	caftaric acid	Phenolic acids	11682.9	2406.3	11399.9	788.3	10466.1	1049.5	11680.3	215.0	11161.3	1452.7	10105.2	1059.5	9911.6	162.5	12101.1	1268.4	9567.9	581.1
m39	fertaric acid	Phenolic acids	1274.2	333.3	1420.2	105.6	1373.5	92.5	1234.6	151.7	1324.9	196.6	1287.1	154.3	1277.9	104.9	1536.0	188.0	1175.8	59.4
m40	resveratrol	Stilbenoid DP1	na	na	75.7	6.9	8.2	5.7	179.7	25.9	68.5	10.2	32.1	5.1	53.3	6.9	96.4	22.9	12.5	0.8
m41	piceid	Stilbenoid DP1	1567.8	113.1	1711.4	270.6	146.8	33.5	3234.5	471.4	2130.8	550.4	618.9	83.1	1697.5	76.6	580.1	300.5	779.9	87.2
m42	pallidol	Stilbenoid DP2	66.2	227.9	137.6	158.5	182.9	219.4	11.5	42.6	63.1	9.1	36.4	12.3	15.6	58.6	32.2	1.2	7.8	10.4
m43	e-viniferin	Stilbenoid DP2	30.1	na	53.9	na	na	na	12.4	20.3	14.3	na	na	na	na	na	na	1.7	3.4	na
